# Supplementary material for: Modeling glioblastoma heterogeneity as a dynamic network of cell states
Source: Mol Syst Biol. 2021 Sep 16;17(9):e10105. doi: 10.15252/msb.202010105 (PMC8444284; doi:10.15252/msb.202010105)
Supplement: Supplementary file 6 — Source Data for Figure 5 [file MSB-17-e10105-s004.zip › Figure5A_sourcedata/GSEA_3017/nosigdb_stateE.GseaPreranked.1622795031093/gsea_report_for_na_pos_1622795031093.html]

Report for na\_pos 1622795031093 [GSEA]

| GS  follow link to MSigDB | GS DETAILS | SIZE | ES | NES | NOM p-val | FDR q-val | FWER p-val | RANK AT MAX | LEADING EDGE || 1 | GAROFANO\_IAVARONE\_GPM\_SUBTYPE | Details ... | 99 | 0.69 | 5.36 | 0.000 | 0.000 | 0.000 | 269 | tags=88%, list=28%, signal=110% |
| 2 | RICHARDS\_NOWAKOWSKI\_SCIENCE\_2017\_U1\_UPREG | Details ... | 112 | 0.53 | 4.17 | 0.000 | 0.000 | 0.000 | 263 | tags=70%, list=28%, signal=85% |
| 3 | RICHARDS\_NOWAKOWSKI\_SCIENCE\_2017\_ORG\_UPREG | Details ... | 159 | 0.49 | 4.12 | 0.000 | 0.000 | 0.000 | 270 | tags=67%, list=29%, signal=78% |
| 4 | RICHARDS\_ZHONG\_NATURE\_2018\_ASTROCYTES\_UPREG | Details ... | 60 | 0.59 | 4.02 | 0.000 | 0.000 | 0.000 | 260 | tags=82%, list=27%, signal=105% |
| 5 | NB\_KI\_CLUSTER18 | Details ... | 21 | 0.80 | 3.84 | 0.000 | 0.000 | 0.000 | 184 | tags=95%, list=19%, signal=116% |
| 6 | VANGRONINGEN\_MES | Details ... | 61 | 0.58 | 3.83 | 0.000 | 0.000 | 0.000 | 263 | tags=79%, list=28%, signal=102% |
| 7 | RICHARDS\_NOWAKOWSKI\_SCIENCE\_2017\_MURAL\_UPREG | Details ... | 77 | 0.53 | 3.83 | 0.000 | 0.000 | 0.000 | 263 | tags=70%, list=28%, signal=89% |
| 8 | OLSEN\_MESENCHYMAL | Details ... | 21 | 0.79 | 3.77 | 0.000 | 0.000 | 0.000 | 113 | tags=81%, list=12%, signal=90% |
| 9 | OLSEN\_SCPLIKE | Details ... | 25 | 0.71 | 3.73 | 0.000 | 0.000 | 0.000 | 127 | tags=72%, list=13%, signal=81% |
| 10 | RICHARDS\_NOWAKOWSKI\_SCIENCE\_2017\_ENDOTHELIAL\_UPREG | Details ... | 120 | 0.46 | 3.71 | 0.000 | 0.000 | 0.000 | 263 | tags=63%, list=28%, signal=77% |
| 11 | RICHARDS\_NOWAKOWSKI\_SCIENCE\_2017\_VRG\_UPREG | Details ... | 79 | 0.51 | 3.70 | 0.000 | 0.000 | 0.000 | 268 | tags=73%, list=28%, signal=94% |
| 12 | RICHARDS\_NOWAKOWSKI\_SCIENCE\_2017\_MGE\_RG1\_UPREG | Details ... | 48 | 0.56 | 3.58 | 0.000 | 0.000 | 0.000 | 263 | tags=79%, list=28%, signal=104% |
| 13 | NB\_KI\_CLUSTER17 | Details ... | 26 | 0.68 | 3.52 | 0.000 | 0.000 | 0.000 | 263 | tags=92%, list=28%, signal=124% |
| 14 | IVY\_CTPAN | Details ... | 24 | 0.69 | 3.47 | 0.000 | 0.000 | 0.000 | 142 | tags=71%, list=15%, signal=81% |
| 15 | SUVA\_MES1\_PMID\_31327527 | Details ... | 16 | 0.79 | 3.44 | 0.000 | 0.000 | 0.000 | 52 | tags=69%, list=5%, signal=72% |
| 16 | RICHARDS\_NOWAKOWSKI\_SCIENCE\_2017\_EN\_V1\_3\_UPREG | Details ... | 87 | 0.44 | 3.39 | 0.000 | 0.000 | 0.000 | 285 | tags=68%, list=30%, signal=88% |
| 17 | RICHARDS\_NOWAKOWSKI\_SCIENCE\_2017\_EN\_PFC2\_UPREG | Details ... | 100 | 0.43 | 3.38 | 0.000 | 0.000 | 0.000 | 285 | tags=66%, list=30%, signal=84% |
| 18 | RICHARDS\_NEFTEL\_CELL\_2019\_MES1 | Details ... | 16 | 0.79 | 3.36 | 0.000 | 0.000 | 0.000 | 52 | tags=69%, list=5%, signal=72% |
| 19 | GANDAL\_AUTISM\_UP\_PMID29439242 | Details ... | 24 | 0.67 | 3.35 | 0.000 | 0.000 | 0.000 | 51 | tags=58%, list=5%, signal=60% |
| 20 | SUVA\_AC\_PMID\_31327527 | Details ... | 15 | 0.77 | 3.27 | 0.000 | 0.000 | 0.000 | 151 | tags=87%, list=16%, signal=101% |
| 21 | RICHARDS\_NOWAKOWSKI\_SCIENCE\_2017\_ASTROCYTE\_UPREG |  | 94 | 0.42 | 3.22 | 0.000 | 0.000 | 0.000 | 241 | tags=62%, list=25%, signal=75% |
| 22 | RICHARDS\_VERHAAK\_CANCERCELL\_2010\_MESENCHYMAL |  | 16 | 0.73 | 3.21 | 0.000 | 0.000 | 0.000 | 263 | tags=100%, list=28%, signal=136% |
| 23 | RICHARDS\_NEFTEL\_CELL\_2019\_AC |  | 15 | 0.77 | 3.18 | 0.000 | 0.000 | 0.000 | 151 | tags=87%, list=16%, signal=101% |
| 24 | DOROSZKO\_U3054MG\_CLUSTER\_2 |  | 45 | 0.50 | 3.18 | 0.000 | 0.000 | 0.000 | 240 | tags=69%, list=25%, signal=88% |
| 25 | LARSSON\_STATE\_6 |  | 26 | 0.58 | 3.08 | 0.000 | 0.000 | 0.000 | 206 | tags=73%, list=22%, signal=91% |
| 26 | RICHARDS\_NOWAKOWSKI\_SCIENCE\_2017\_TRG\_UPREG |  | 155 | 0.36 | 3.05 | 0.000 | 0.000 | 0.000 | 252 | tags=54%, list=27%, signal=62% |
| 27 | RICHARDS\_VERHAAK\_CANCERCELL\_2010\_CLASSICAL |  | 24 | 0.57 | 2.99 | 0.000 | 0.000 | 0.000 | 258 | tags=83%, list=27%, signal=112% |
| 28 | RICHARDS\_NOWAKOWSKI\_SCIENCE\_2017\_EN\_V1\_2\_UPREG |  | 86 | 0.38 | 2.97 | 0.000 | 0.000 | 0.000 | 266 | tags=59%, list=28%, signal=75% |
| 29 | DOROSZKO\_PERIVASCULAR\_INVASION |  | 16 | 0.67 | 2.91 | 0.000 | 0.000 | 0.000 | 180 | tags=75%, list=19%, signal=91% |
| 30 | RICHARDS\_NOWAKOWSKI\_SCIENCE\_2017\_MICROGLIA\_UPREG |  | 65 | 0.42 | 2.87 | 0.000 | 0.000 | 0.000 | 207 | tags=58%, list=22%, signal=70% |
| 31 | RICHARDS\_NOWAKOWSKI\_SCIENCE\_2017\_EN\_PFC3\_UPREG |  | 44 | 0.47 | 2.84 | 0.000 | 0.000 | 0.000 | 285 | tags=70%, list=30%, signal=96% |
| 32 | NB\_KI\_CLUSTER23 |  | 19 | 0.59 | 2.76 | 0.000 | 0.000 | 0.000 | 259 | tags=84%, list=27%, signal=114% |
| 33 | RICHARDS\_NOWAKOWSKI\_SCIENCE\_2017\_EN\_PFC1\_UPREG |  | 95 | 0.34 | 2.66 | 0.000 | 0.000 | 0.000 | 223 | tags=52%, list=24%, signal=61% |
| 34 | GANDAL\_SCHIZOPHRENIA\_UP\_PMID29439242 |  | 27 | 0.50 | 2.65 | 0.000 | 0.000 | 0.000 | 261 | tags=78%, list=28%, signal=104% |
| 35 | RICHARDS\_NOWAKOWSKI\_SCIENCE\_2017\_EN\_V1\_1\_UPREG |  | 82 | 0.35 | 2.65 | 0.000 | 0.000 | 0.000 | 316 | tags=65%, list=33%, signal=89% |
| 36 | DOROSZKO\_U3180MG\_CLUSTER\_2 |  | 50 | 0.42 | 2.65 | 0.000 | 0.000 | 0.000 | 244 | tags=62%, list=26%, signal=79% |
| 37 | RICHARDS\_CAHOY\_JNEUROSCI\_2015\_ASTRO\_IN\_VIVO |  | 121 | 0.33 | 2.63 | 0.000 | 0.000 | 0.000 | 266 | tags=56%, list=28%, signal=68% |
| 38 | DOROSZKO\_U3054MG\_CLUSTER\_10 |  | 23 | 0.51 | 2.58 | 0.000 | 0.000 | 0.001 | 268 | tags=74%, list=28%, signal=101% |
| 39 | LARSSON\_STATE\_1 |  | 27 | 0.49 | 2.54 | 0.000 | 0.000 | 0.001 | 261 | tags=78%, list=28%, signal=104% |
| 40 | RICHARDS\_CAHOY\_JNEUROSCI\_2011\_ASTRO\_MATURE |  | 39 | 0.42 | 2.45 | 0.000 | 0.000 | 0.001 | 257 | tags=64%, list=27%, signal=84% |
| 41 | DOROSZKO\_U3013MG\_CLUSTER\_3 |  | 17 | 0.52 | 2.26 | 0.000 | 0.001 | 0.006 | 40 | tags=35%, list=4%, signal=36% |
| 42 | RICHARDS\_CAHOY\_JNEUROSCI\_2008\_ASTROCYTE |  | 166 | 0.26 | 2.25 | 0.000 | 0.001 | 0.007 | 266 | tags=51%, list=28%, signal=58% |
| 43 | DOROSZKO\_U3054MG\_CLUSTER\_5 |  | 16 | 0.49 | 2.04 | 0.000 | 0.004 | 0.046 | 239 | tags=75%, list=25%, signal=99% |
| 44 | RICHARDS\_CAHOY\_JNEUROSCI\_2013\_OL\_MYEL |  | 88 | 0.25 | 1.92 | 0.000 | 0.007 | 0.082 | 266 | tags=51%, list=28%, signal=65% |
| 45 | RICHARDS\_ZHONG\_NATURE\_2018\_MICROGLIA\_UPREG |  | 22 | 0.38 | 1.88 | 0.000 | 0.010 | 0.108 | 206 | tags=64%, list=22%, signal=79% |
| 46 | RICHARDS\_NOWAKOWSKI\_SCIENCE\_2017\_NEN\_EARLY2\_UPREG |  | 43 | 0.30 | 1.86 | 0.000 | 0.010 | 0.115 | 255 | tags=56%, list=27%, signal=73% |
| 47 | RICHARDS\_NOWAKOWSKI\_SCIENCE\_2017\_CHOROID\_UPREG |  | 61 | 0.27 | 1.80 | 0.000 | 0.016 | 0.182 | 125 | tags=31%, list=13%, signal=34% |
| 48 | RICHARDS\_CAHOY\_JNEUROSCI\_2009\_NEURON |  | 74 | 0.25 | 1.79 | 0.000 | 0.016 | 0.186 | 255 | tags=49%, list=27%, signal=61% |
| 49 | RICHARDS\_CAHOY\_JNEUROSCI\_2014\_MOG\_POS |  | 59 | 0.27 | 1.79 | 0.000 | 0.016 | 0.188 | 217 | tags=44%, list=23%, signal=54% |
| 50 | RICHARDS\_NOWAKOWSKI\_SCIENCE\_2017\_GLYC\_UPREG |  | 53 | 0.27 | 1.74 | 0.006 | 0.021 | 0.245 | 411 | tags=72%, list=43%, signal=120% |
| 51 | NB\_KI\_CLUSTER2 |  | 33 | 0.30 | 1.69 | 0.010 | 0.026 | 0.294 | 411 | tags=76%, list=43%, signal=129% |
| 52 | DOROSZKO\_U3054MG\_CLUSTER\_9 |  | 36 | 0.29 | 1.68 | 0.038 | 0.028 | 0.315 | 243 | tags=47%, list=26%, signal=61% |
| 53 | ALMSTEDT\_VGR\_DIFF |  | 125 | 0.20 | 1.61 | 0.000 | 0.040 | 0.447 | 95 | tags=22%, list=10%, signal=21% |
| 54 | NB\_KI\_CLUSTER16 |  | 18 | 0.36 | 1.60 | 0.041 | 0.042 | 0.464 | 175 | tags=50%, list=18%, signal=60% |
| 55 | GANDAL\_BIPOLAR\_UP\_PMID29439242 |  | 18 | 0.34 | 1.52 | 0.079 | 0.062 | 0.621 | 260 | tags=67%, list=27%, signal=90% |
| 56 | RICHARDS\_NOWAKOWSKI\_SCIENCE\_2017\_IN\_CTX\_CGE2\_UPREG |  | 35 | 0.26 | 1.47 | 0.039 | 0.086 | 0.742 | 241 | tags=49%, list=25%, signal=63% |
| 57 | RICHARDS\_NOWAKOWSKI\_SCIENCE\_2017\_NEN\_EARLY1\_UPREG |  | 63 | 0.21 | 1.46 | 0.037 | 0.087 | 0.752 | 322 | tags=56%, list=34%, signal=79% |
| 58 | LARSSON\_STATE\_5 |  | 16 | 0.31 | 1.32 | 0.158 | 0.171 | 0.948 | 168 | tags=50%, list=18%, signal=60% |
| 59 | DOROSZKO\_U3054MG\_CLUSTER\_3 |  | 25 | 0.23 | 1.22 | 0.220 | 0.264 | 0.989 | 575 | tags=88%, list=61%, signal=218% |
| 60 | NB\_KI\_CLUSTER6 |  | 32 | 0.22 | 1.20 | 0.214 | 0.277 | 0.992 | 746 | tags=100%, list=79%, signal=455% |
| 61 | RICHARDS\_NOWAKOWSKI\_SCIENCE\_2017\_NEN\_LATE\_UPREG |  | 34 | 0.21 | 1.20 | 0.262 | 0.273 | 0.992 | 264 | tags=47%, list=28%, signal=63% |
| 62 | NB\_KI\_CLUSTER14 |  | 20 | 0.24 | 1.18 | 0.246 | 0.292 | 0.994 | 282 | tags=60%, list=30%, signal=84% |
| 63 | RICHARDS\_NOWAKOWSKI\_SCIENCE\_2017\_IN\_CTX\_MGE2\_UPREG |  | 27 | 0.22 | 1.15 | 0.245 | 0.332 | 0.996 | 265 | tags=52%, list=28%, signal=70% |
| 64 | RICHARDS\_NOWAKOWSKI\_SCIENCE\_2017\_IPC\_NEN1\_UPREG |  | 30 | 0.21 | 1.15 | 0.301 | 0.327 | 0.996 | 198 | tags=43%, list=21%, signal=53% |
| 65 | NB\_KI\_CLUSTER4 |  | 19 | 0.24 | 1.10 | 0.314 | 0.381 | 0.998 | 238 | tags=47%, list=25%, signal=62% |
| 66 | RICHARDS\_NOWAKOWSKI\_SCIENCE\_2017\_IN\_CTX\_CGE1\_UPREG |  | 35 | 0.19 | 1.08 | 0.374 | 0.410 | 0.998 | 775 | tags=100%, list=82%, signal=530% |
| 67 | RICHARDS\_NOWAKOWSKI\_SCIENCE\_2017\_IN\_CTX\_MGE1\_UPREG |  | 39 | 0.17 | 1.05 | 0.417 | 0.448 | 0.998 | 791 | tags=100%, list=84%, signal=582% |
| 68 | GAROFANO\_IAVARONE\_NEU\_SUBTYPE |  | 25 | 0.20 | 1.01 | 0.427 | 0.513 | 0.999 | 201 | tags=40%, list=21%, signal=49% |
| 69 | DOROSZKO\_U3054MG\_CLUSTER\_8 |  | 35 | 0.16 | 0.92 | 0.608 | 0.655 | 1.000 | 797 | tags=100%, list=84%, signal=608% |
| 70 | RICHARDS\_NOWAKOWSKI\_SCIENCE\_2017\_NIN5\_UPREG |  | 36 | 0.16 | 0.90 | 0.560 | 0.672 | 1.000 | 802 | tags=100%, list=85%, signal=628% |
| 71 | RICHARDS\_NOWAKOWSKI\_SCIENCE\_2017\_OPC\_UPREG |  | 64 | 0.13 | 0.90 | 0.647 | 0.670 | 1.000 | 133 | tags=22%, list=14%, signal=24% |
| 72 | RICHARDS\_NOWAKOWSKI\_SCIENCE\_2017\_IPC\_NEN3\_UPREG |  | 31 | 0.16 | 0.87 | 0.635 | 0.704 | 1.000 | 243 | tags=45%, list=26%, signal=59% |
| 73 | RICHARDS\_CAHOY\_JNEUROSCI\_2008\_OLIGODENDROCYTE |  | 124 | 0.11 | 0.84 | 0.803 | 0.745 | 1.000 | 244 | tags=36%, list=26%, signal=42% |
| 74 | RICHARDS\_NOWAKOWSKI\_SCIENCE\_2017\_IN\_STR\_UPREG |  | 17 | 0.19 | 0.82 | 0.697 | 0.758 | 1.000 | 771 | tags=100%, list=81%, signal=528% |
| 75 | RICHARDS\_ZHONG\_NATURE\_2018\_OPC\_UPREG |  | 22 | 0.16 | 0.76 | 0.784 | 0.828 | 1.000 | 105 | tags=23%, list=11%, signal=25% |
| 76 | RICHARDS\_NOWAKOWSKI\_SCIENCE\_2017\_NIN3\_UPREG |  | 20 | 0.15 | 0.71 | 0.816 | 0.882 | 1.000 | 169 | tags=30%, list=18%, signal=36% |
| 77 | RICHARDS\_NOWAKOWSKI\_SCIENCE\_2017\_NIN2\_UPREG |  | 51 | 0.10 | 0.62 | 0.934 | 0.946 | 1.000 | 778 | tags=96%, list=82%, signal=509% |
| 78 | RICHARDS\_NOWAKOWSKI\_SCIENCE\_2017\_U4\_UPREG |  | 118 | 0.07 | 0.53 | 0.984 | 0.979 | 1.000 | 79 | tags=11%, list=8%, signal=11% |
Table: Gene sets enriched in phenotype **na **[plain text format]****
